# Cross-trait analysis reveals shared genetic architecture between mental health and cardiovascular traits

**DOI:** 10.1016/j.gmg.2026.100122

**Published:** 2026-07-24

**Authors:** Quanting Yin, Jun Zhao, Pengcheng Yi, Chunhua Yang, Jia Mi, Fuyi Xu, Geng Tian, Li Shang, Qingxia He

**Affiliations:** a Shandong Technology Innovation Center of Molecular Targeting and Intelligent Diagnosis and Treatment, School of Pharmacy Shandong Medical And Pharmaceutical University, Yantai, Shandong 264003, China; b Shandong Province Key Laboratory of Complex Medical Intelligence and Aging, Shandong Medical and Pharmaceutical University, Yantai 264003, China; c Department of Pathology, Shandong Medical and Pharmaceutical University Hospital, Binzhou 256699, China; d Yantai Affiliated Hospital of Shandong Medical and Pharmaceutical University, Yantai, Shandong 264000, China; e School of Special Education and Rehabilitation Shandong Medical and Pharmaceutical University, Yantai, Shandong 264003, China

**Keywords:** Cardiovascular system, Mental health, Mendelian randomization

## Abstract

**Background:**

The association between cardiovascular disease (CVD) and mental health (MH) is well established. However, the shared genetic architecture and underlying causal relationships between these conditions remain insufficiently understood.

**Material and methods:**

Summary statistics from Genome-wide association studies (GWAS) for 8 MH traits and 10 cardiovascular system (CVS) traits were used in the analyses. LDSC regression was applied to estimate heritability and genetic correlations. Bidirectional Mendelian randomization (MR) analysis was conducted to explore potential causal relationships between MH and CVS traits. In addition, Summary data-based Mendelian randomization (SMR) analysis was used to identify gene expression in circulating causally associated with both MH and CVS traits. Functional annotation of genes was further explored by querying gene information across multiple databases.

**Results:**

LDSC identified five cross-trait genetic correlations that survived Bonferroni correction, with nine additional pairs reaching false discovery rate significance. Bidirectional causality between DBP and anxiety, and unidirectional links between DBP-NM, SBP-anxiety, anxiety-angina, and NM-CAD/myocardial infarction. SMR analysis further identified 12 shared genes that regulate both MH and CVS traits. Notably, CNNM2 was implicated across transcriptional levels, mouse knockout models, and pathogenic mutations, with associations to both cardiovascular and neurological phenotypes. Additionally, RGS6 and GAB2 simultaneously influence both anxiety and blood pressure, and ZCCHC14 exhibits pleiotropic effects on heart rate variability and blood vessels.

**Conclusion:**

Our study provides evidence for a shared genetic basis and suggests potential causal relationships between CVS and MH traits. This highlights the intricate interplay between genetic factors, mental well-being, and cardiovascular health, underscoring the value of further mechanistic and experimental research to clarify how these shared pathways might eventually inform clinical strategies.

## Introduction

Cardiovascular diseases (CVD) remain leading contributors to global morbidity and mortality. While traditional risk factors like smoking and hypertension are well-established, the significant impact of mental health (MH) on CVD risk deserves equal attention [1]. Previous studies have demonstrated that psychological states significantly influence CVD risk, reinforcing the association between CVD and various psychological conditions, such as depression and anxiety [2], [3].

The observed association between CVD and MH can be partly explained by shared genetic underpinnings [3], [4], [5], [6]. For example, there is significant genetic overlap observed between schizophrenia and the risk factors associated with CVD [5]. Additionally, a recent study found that loneliness shares several genetic loci with CVD risk factors, including systolic blood pressure (SBP) (9 loci), diastolic blood pressure (DBP) (4 loci), and coronary artery disease (CAD) (12 loci) [7]. Furthermore, A study have found a significant genetic correlation between CVD and schizophrenia, with 842 potential pleiotropic SNPs shared between the two [8]. Therefore, understanding the genetic links between MH and CVD could provide insights into their shared etiology and help guide future research into integrated approaches for managing these co-occurring conditions.

Large-scale GWAS data provide a valuable resource for exploring pleiotropic genetic architectures across complex traits. For example, Linkage Disequilibrium Score Regression (LDSC), introduced by Bulik-Sullivan et al., utilizes GWAS summary statistics to estimate SNP-based genetic correlations between traits, offering insights into shared genetic underpinnings [9]. Building on this, Mendelian Randomization (MR) leverages genetic variants as instrumental variables (IVs) to infer causal relationships between modifiable exposures and health outcomes, mimicking a natural randomized controlled trial by exploiting the random assortment of alleles during gamete formation [10]. Furthermore, integrating eQTL data with GWAS summary statistics through methods like SMR enables the identification of causal pathways linking gene expression to complex traits [11].

In this study, we applied these methods to explore the shared genetic architecture between the MH and CVS. LDSC was used to estimate genetic correlations and bidirectional MR to investigate potential causal relationships between 8 MH and 10 CVS traits in European populations. Furthermore, we employed SMR to examine the influence of gene expression in circulating on both MH and CVS traits. Together, these approaches offer new insights into the genetic links between cardiovascular health and mental well-being, paving the way for future research into understanding these interconnected conditions.

## Materials and methods

### GWAS statistics

In this study, GWAS summary statistics for 8 MH traits, including Attention-Deficit/Hyperactivity Disorder (ADHD), Anorexia Nervosa (AN), Anxiety, Bipolar Disorder (BD), Neuroticism measurement (NM), Obsessive-Compulsive Disorder (OCD), Schizophrenia, and Unipolar Depression (UD) were obtained from GWAS data available in the IEU Open GWAS (https://gwas.mrcieu.ac.uk/) and GWAS Catalog (https://www.ebi.ac.uk/gwas/) (Table 1). GWAS summary statistics for 10 CVS traits, including Angina Pectoris (AP), Acute Pericarditis (APE), Atrial Fibrillation (AF), Coronary Artery Disease (CAD), Diastolic Blood Pressure (DBP), Systolic Blood Pressure (SBP), Hypertrophic Cardiomyopathy (HCM), Myocardial Infarction (MI), Heart Failure (HF), and Heart Valve Disorders (HV), were sourced from the IEU Open GWAS and the Mendeley Data (https://data.mendeley.com/). These data are all from European populations (Table 1).

**Table 1:** Overview of cardiovascular system traits and mental health traits.

**Traits**	**Abbreviation**	**ID**	**Source**	**Sample size**	**Ancestry**
attention-deficit/hyperactivity disorder	ADHD	GCST005362	GWAScatlog	32,102 (case: 14,154)	European (Denmark, Germany, etc.)
anorexia nervosa	AN	ieu-a−1186	IEU open GWAS	14,477 (case: 3495)	European
anxiety	-	GCST90041879	GWAScatlog	444,404 (case: 251,982)	European
bipolar disorder	BD	ieu-b−5110	IEU open GWAS	413,466 (case: 41,917)	European
neuroticism measurement	NM	GCST006940	GWAScatlog	380,506	European
obsessive-compulsive disorder	OCD	ieu-a−1189	IEU open GWAS	33,925 (case: 26,888)	European (NR)
schizophrenia	-	GCST90043704	GWAScatlog	456,348 (case: 376)	European (U.K.)
unipolar depression	UD	GCST90043709	GWAScatlog	456,348 (case: 376)	European (U.K.)
angina pectoris	AP	ukb-a−532	IEU open GWAS	337,199 (case: 4837)	European
Acute Pericarditis	APE	ukb-a−535	IEU open GWAS	337,199 (case: 107)	European
Atrial Fibrillation	AF	29892015	CVDKP Website	537,409 (case: 55,114)	European
Coronary Artery Disease	CAD	29212778	Mendeley Data	296,525 (case: 34,541)	European
Diastolic Blood Pressure	DBP	ieu-b−39	IEU open GWAS	757,601	European
Systolic Blood Pressure	SBP	ieu-b−38	IEU open GWAS	757,601	European
Hypertrophic Cardiomyopathy	HCM	ukb-a−114	IEU open GWAS	337,159 (case: 71)	European
Myocardial Infarction	MI	ukb-d-I9_ MI	IEU open GWAS	361,194 (case: 7018)	European
Heart Failure	HF	ebi-a-GCST009541	IEU open GWAS	977,323 (case: 47,309)	European
Heart valve problem	HV	ukb-b−18578	IEU open GWAS	462,933 (case: 3162)	European

### Genetic correlation analysis

We computed heritability and genetic correlations between MH and CVS traits by LDSC [12], [9]. SNPs located within the major histocompatibility complex (MHC) region (chromosome 6: 28,477,797–33,448,354), exhibiting strand ambiguity (e.g., A/T and G/C SNPs), or with a minor allele frequency (MAF) below 0.01, were excluded from the LDSC analysis. The linkage disequilibrium (LD) reference panel employed in this study was sourced from the European Ancestry 1000 Genomes dataset. A total of 80 cross-trait genetic correlations were tested. The primary significance threshold was defined using Bonferroni correction as P < 6.25 × 10^−4^. As a complementary analysis, the Benjamini–Hochberg false discovery rate was calculated across the 80 tests. Associations with q < 0.05 that did not meet the Bonferroni threshold were classified as FDR-significant. Associations with nominal P < 0.05 that did not survive either multiple-testing correction were considered exploratory.

### MR analysis

We first selected SNPs significantly associated with MH and CVS traits from GWAS data as IVs, applying a genome-wide significance threshold of P < 5 × 10⁻⁸. Concurrently, LD among the selected SNPs was assessed using the PLINK clustering method (R² = 0.001, clustering distance = 1000 kb) [13]. The F-statistic was calculated for each SNP, and those with an F-statistic below 10 were excluded to reduce the risk of weak instrument bias. These analyses were conducted using the TwoSampleMR package. To enhance the accuracy and robustness of genetic instruments, a heterogeneity test was conducted to identify and adjust for outliers. Cochran’s Q test was applied to fit the inverse variance-weighted (IVW) model in the presence of horizontal pleiotropy effects [14], while Rucker’s Q′ test was employed for MR-Egger model fitting to account for directional pleiotropy effects [15].

Additionally, we conducted bidirectional MR analysis, specifically investigating the causal relationship between MH and CVS traits. For each potential causal direction, we aggregated MR estimates through IVW meta-analysis, with a significant level set at P < 0.05. This involved a weighted regression of SNP-outcome effects on SNP-exposure effects with the constraint of a zero intercept. Moreover, we employed MR-Egger, weighted median, Simple mode, and weighting mode approaches for supplementary analyses. The MR-Egger method can determine if genetic variants have pleiotropic effects on the outcome that, on average, deviate from zero (indicating directional pleiotropy) [16]. The weighted median provides an accurate estimate under the condition that a minimum of 50% of IVs are valid and it is complementary to the recently proposed MR-Egger regression method [17]. While the simple mode may lack the power of IVW, it offers robustness against pleiotropy [18].

Furthermore, we conducted assessments for directional horizontal pleiotropy using MR-Egger intercept tests [19], [20]. In Egger regression, a non-zero intercept (p < 0.05) indicates the presence of pleiotropy. Next, we conducted leave-one-out analysis to determine if the causal association was significantly influenced by any single SNP.

### Summary data-based Mendelian randomization (SMR) analysis and HEIDI test

The SMR version 1.3.1 [21] was used to identify gene expression in circulating that were causally associated with both MH and CVS traits. This method integrates summary-level data from GWAS and eQTL studies by applying MR principles, allowing us to focus on functionally significant genes within GWAS loci. Specifically, we used eQTL data from the eQTLgen consortium (https://www.eqtlgen.org/phase1.html), which includes 10,317 trait-associated SNPs from 31,684 blood samples [22]. In this analysis, we focused on cis-eQTLs, which are regulatory variants linked to the expression of nearby genes. To ensure that the associations between gene expression and traits were not driven by linkage, we applied the HEIDI (Heterogeneity in Dependent Instruments) test, a key component of the SMR approach. Associations with HEIDI P < 0.05 were considered potentially attributable to linkage or heterogeneity and were excluded, whereas associations with HEIDI P > 0.05 were retained as lacking evidence against a shared causal variant.

### Exploration of gene function

For the genes identified through SMR, comprehensive searches were conducted across several relevant databases to obtain additional functional annotations and validate the gene-trait associations. Data from Transcriptome-Wide Association Studies (TWAS) [23] were queried to investigate the relationship between gene expression and the traits of interest. The International Mouse Phenotyping Consortium (IMPC) [24] database was accessed to examine mouse models with knockout or knockdown mutations of the selected genes, assessing the resulting phenotypic outcomes to confirm their functional roles in disease. Additionally, the Mouse Genome Informatics (MGI) [25]database was searched for detailed genetic, phenotypic, and molecular information on the candidate genes, including known alleles and phenotypes associated with mouse models.

## Results

### Study overview

We initially collected GWAS data for 8 MH traits and 10 CVS traits (Table 1), followed by a global genetic correlation analysis. Trait pairs showing significant genetic correlations were then subjected to bidirectional MR analysis. Subsequently, eQTL data from circulating blood to perform SMR analysis. Finally, functional exploration of genes associated with these causal trait pairs was conducted by querying TWAS, IMPC,and MGI to gather insights into their roles and potential involvement in the observed traits (Fig. 1).

**Fig. 1:**
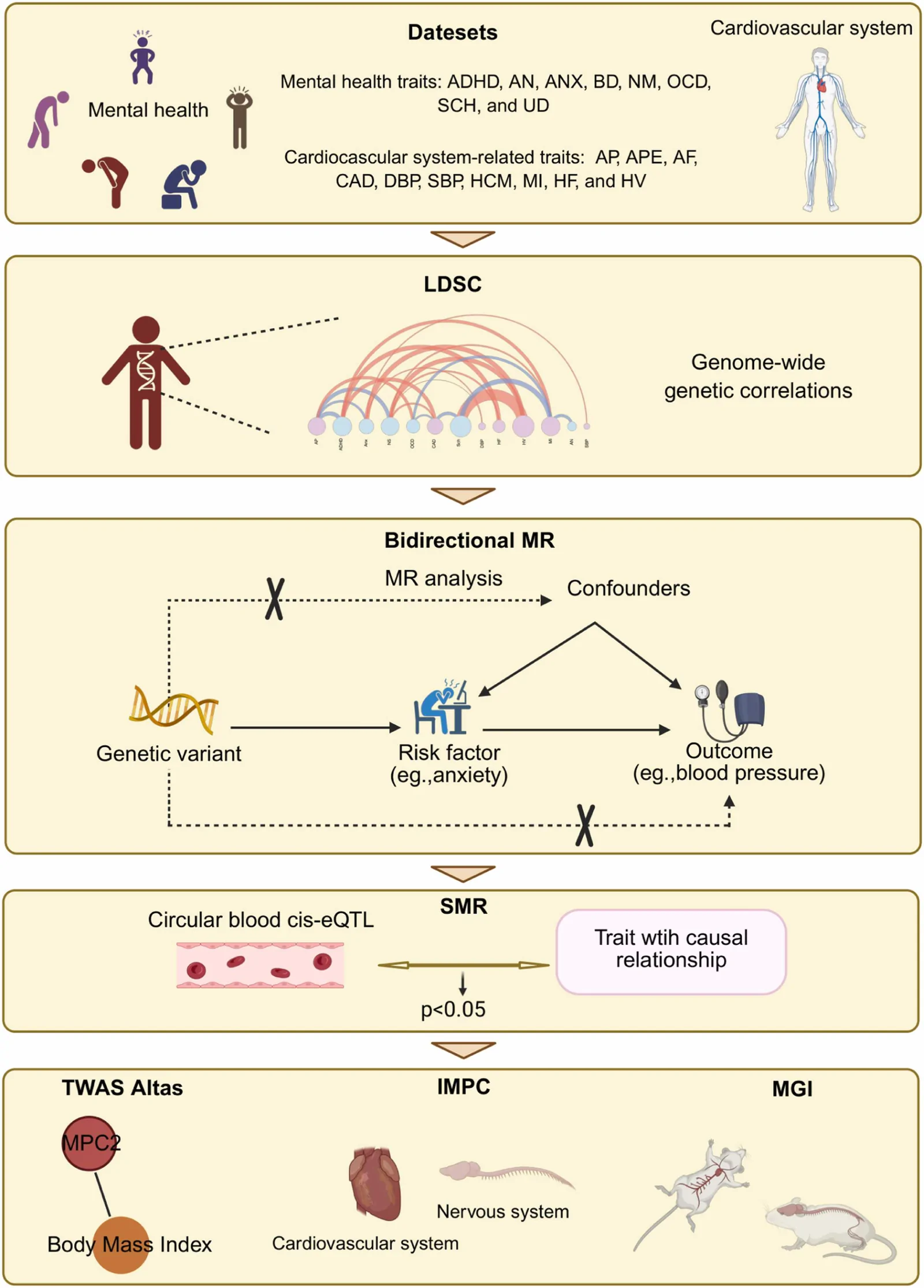
Overview of the study workflow, starting with GWAS data collection, followed by genetic correlation analysis, bidirectional MR, SMR analysis, and functional gene exploration.

### Genetic correlation

LDSC analysis estimated the heritability (*h*^*2*^) of MH and CVS traits, which ranged from 0.002 to 0.26. AN exhibited the highest heritability (*h*^*2*^ = 0.26), closely followed by ADHD (*h*^*2*^ = 0.25). The heritability estimates for SBP and DBP were both approximately 0.14.

To characterize the shared genetic architecture between CVS and MH traits, we conducted bivariate LDSC analyses across 80 trait pairs (10 CVS × 8 MH). At the Bonferroni-corrected threshold (P < 6.25 × 10⁻⁴), five trait pairs were significant (Fig. 2). CAD showed the strongest overlap with mental health traits, with significant positive correlations with ADHD (rg = 0.283, P = 6.05 × 10⁻¹²) and NM (rg = 0.137, P = 7.93 × 10⁻¹⁰). Heart failure and myocardial infarction each showed significant positive genetic correlations with ADHD (HF: rg = 0.333, P = 2.46 × 10⁻⁵; MI: rg = 0.241, P = 4.00 × 10⁻⁴), suggesting that the genetic overlap with ADHD is not limited to CAD. Angina pectoris (AP) showed a significant negative genetic correlation with NM (rg = −0.290, P = 3.56 × 10⁻⁷). Applying the less conservative FDR correction (q < 0.05), nine additional pairs reached significance, expanding the total to 14 significant associations (Fig. 2). AP showed further negative genetic correlations with ADHD (rg = −0.302, P = 1.7 × 10⁻³) and anxiety (rg = −0.177, P = 6.5 × 10⁻³). Blood pressure traits showed positive genetic correlations with anxiety (DBP: rg = 0.081, P = 1.1 × 10⁻³; SBP: rg = 0.079, P = 4.7 × 10⁻³). HF showed an additional positive genetic correlation with NM (rg = 0.131, P = 2.5 × 10⁻³), and MI showed a negative genetic correlation with anorexia nervosa (rg = −0.225, P = 4.1 × 10⁻³). The remaining 7 nominally significant pairs (P < 0.05) did not survive FDR correction.

**Fig. 2:**
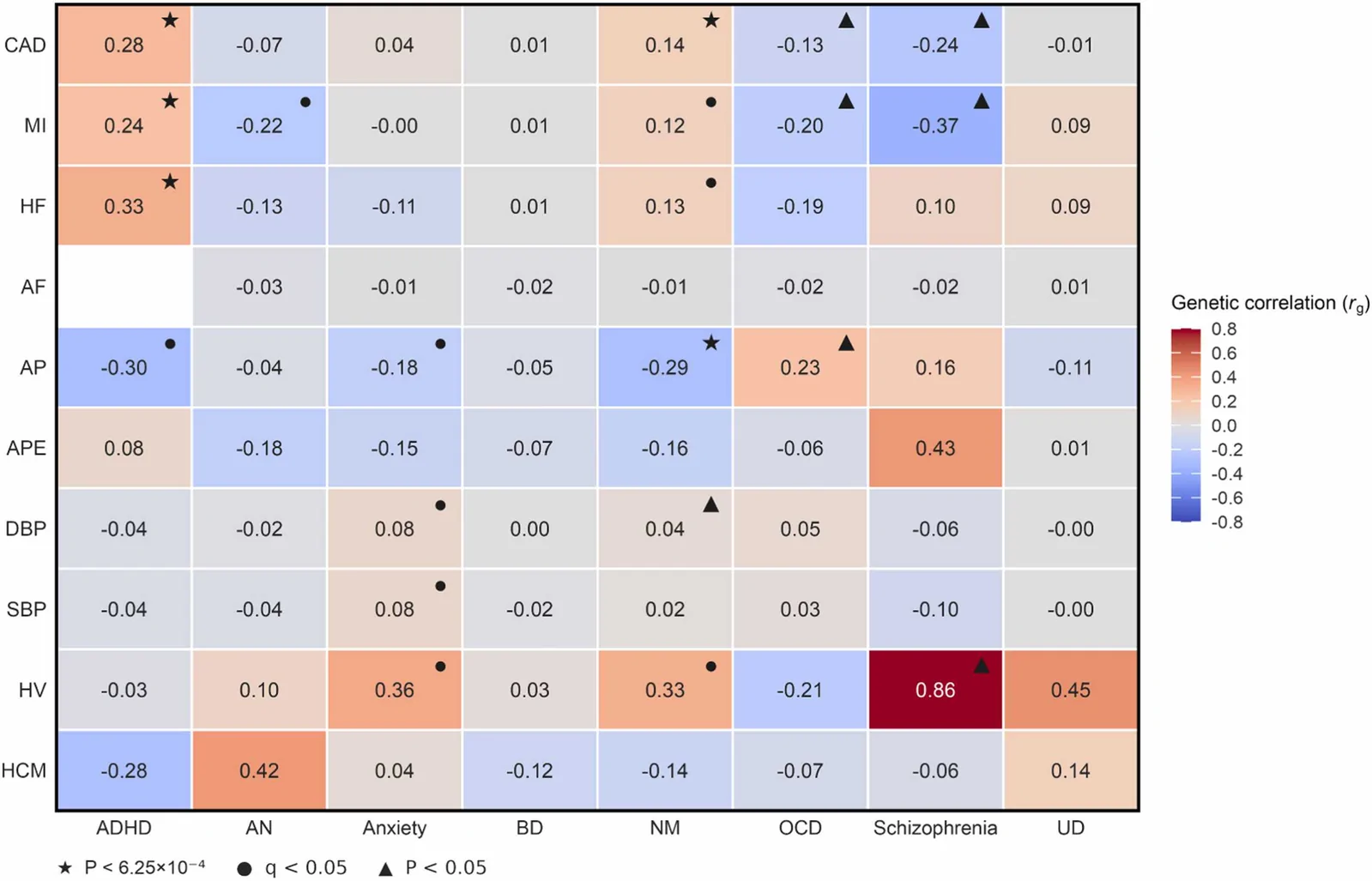
**Genetic correlations between cardiovascular and mental health traits estimated by LDSC.** Numbers indicate genetic correlation coefficients; red and blue represent positive and negative correlations, respectively. Stars indicate Bonferroni-significant associations, circles indicate FDR-significant associations, and triangles indicate nominal associations.

### MR analysis

To obtain robust and reliable IVs, we adhered to the following three key assumptions of the MR approach: (1) the genetic variants (IVs) must be strongly associated with the exposure; (2) the genetic variation should be independent of confounding factors; and (3) the genetic variation should affect the outcome only through the exposure, not through other pathways [26]. We selected IVs strongly associated with the exposure, followed by linkage disequilibrium (LD) pruning. The strength of the IVs was estimated using the F-statistic, which was greater than 30 for both forward and reverse MR phenotypic pairs. Heterogeneity tests were performed, and outliers were removed. Details on the number of SNPs for all phenotypes are provided in supplementary table 1

In the forward MR analyses, we identified 3 causal relationships between blood pressure and MH traits. As shown in Fig. 3, an elevated risk of DBP was associated with an increase in the NM (OR =1.0027, 95%CI of 1.001–1.004, P = 3 ×10^−4^) and a heightened risk of anxiety (OR = 1.0081, 95%CI of 1.005–1.010, P = 2.09 × 10^−9^). And Higher SBP risk was associated with increased anxiety (OR = 1.0038, 95%CI of 1.002–1.005, P = 3.70 × 10^−6^).

**Fig. 3:**
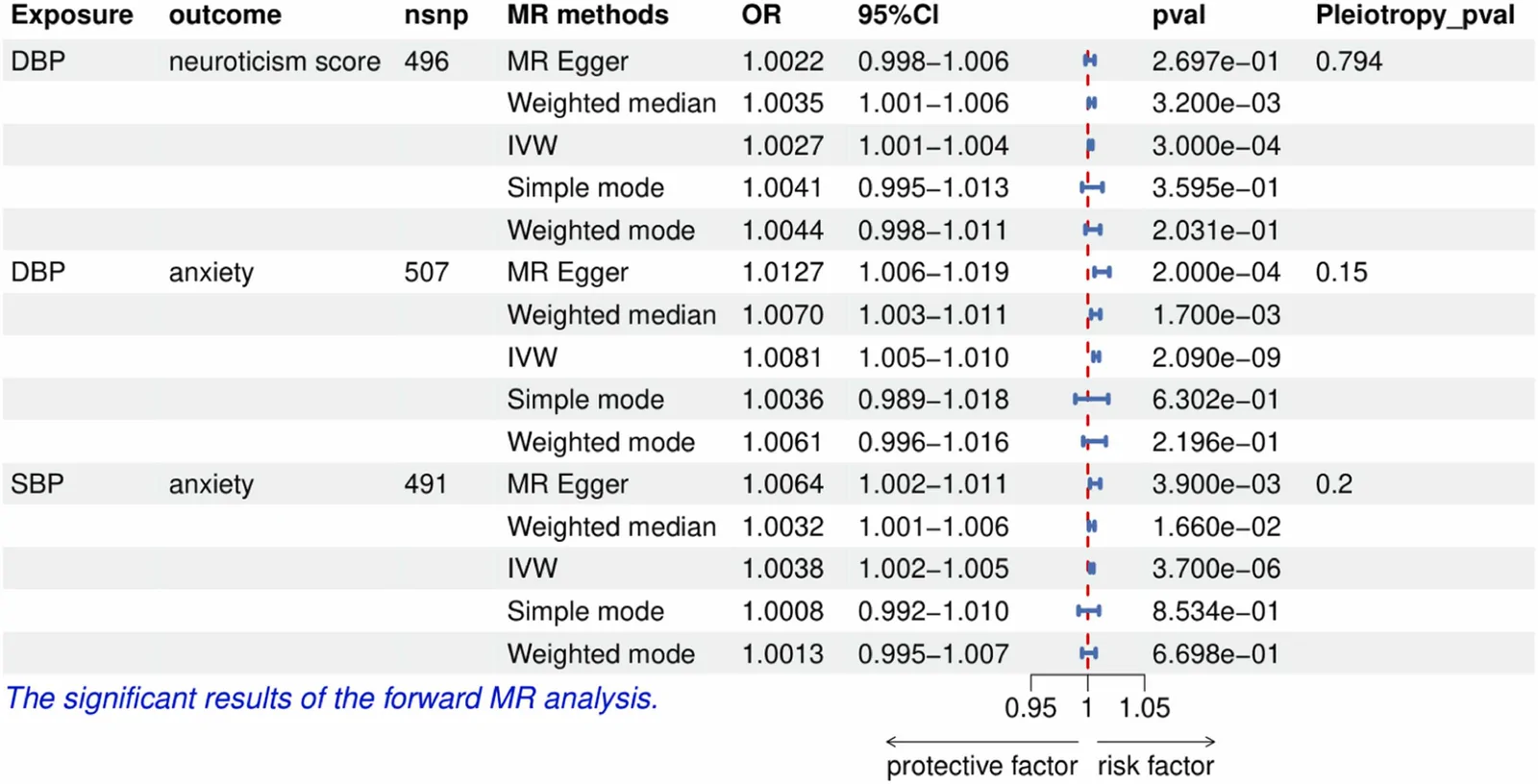
**Forest plot of the forward MR analysis showing.** Causal estimates were obtained using five two-sample MR methods: MR-Egger, Weighted median, IVW, Simple mode, and Weighted mode. A pleiotropy p-value greater than 0.05 was considered indicative of the absence of substantial pleiotropic effects.

To further investigate the causal relationships between MH and CVS traits, reverse MR analysis was conducted. As shown in Fig. 4, a 1 standard deviation (s.d.) increase in the NM was associated with a 44.84% higher odds of CAD (OR = 1.4484, 95%CI= 1.293–1.623, P = 1.64 × 10⁻¹⁰). Additionally, a 1 s.d. increase in the NM was associated with a 0.56% higher odds of MI (OR = 1.0056, 95%CI = 1.000–1.011, P = 0.0236). Moreover, a 1 sd. increase in anxiety was tied to a 48.97% higher odds of DBP (OR = 1.4897, 95%CI = 1.022–2.172, P = 0.0382).

**Fig. 4:**
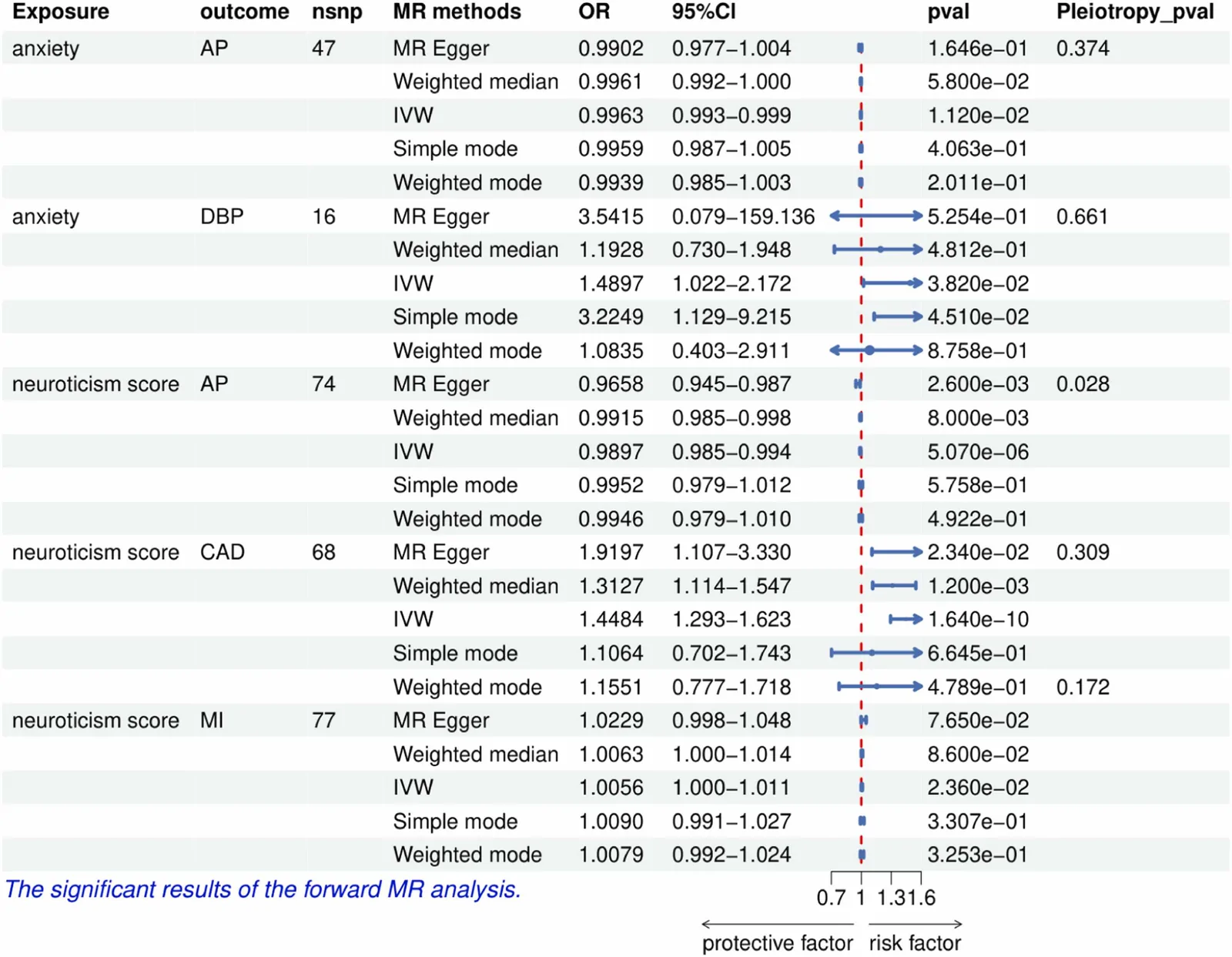
**Forest plot of the reverse MR analysis.** Causal effects were estimated using five two-sample MR methods (MR-Egger, Weighted median, IVW, Simple mode, and Weighted mode.). A pleiotropy p-value exceeding 0.05 was deemed indicative of the absence of substantial pleiotropic effects.

We performed sensitivity analysis to confirm the potential causal relationships derived from MR. First, the leave-one-out analysis suggested that no single SNP was influencing the causal estimates (Supplement Figure 1). Second, the pleiotropy test was conducted (Fig. 3, Fig. 4**)**. It is evident that only NM and AP exhibit multicollinearity (Pleiotropy_pval = 0.02).

### SMR analysis

To investigate whether shared genetic loci underlie the causal trait pairs identified through MR analyses, we performed a SMR analysis. This analysis led to the identification of several genes that are potentially causally associated with both MH and CVS traits including anxiety NM, DBP, SBP, and CAD.

Genes associated both with anxiety and CVS including GAB2, RGS6, FBXO31, MAP1LC3B, TRIM65, ZCCHC14, BCKDK, KAT8, LINC02210, SAE1, and ATXN1L. Notably, GAB2 and RGS6 demonstrated cross-system pleiotropy: GAB2 exerted protective effects against anxiety (OR=0.85), DBP (OR=0.46), and SBP (OR=0.42), whereas RGS6 displayed risk-enhancing effects for anxiety (OR=1.08) but protective associations with DBP (OR=0.53) and SBP (OR=0.28).

Furthermore, FBXO31, MAP1LC3B, TRIM65, and ZCCHC14 exhibited tri-trait associations within the anxiety-DBP-NM network. Specifically, FBXO31, MAP1LC3B, and ZCCHC14 showed risk effects on the CVS metabolic indicator DBP (OR range=1.22–1.38), while conferring protection on MH anxiety (OR=0.93–0.97) and NM traits. Conversely, TRIM65 consistently acted as a risk factor across all three traits (OR range=1.03–1.28).

The anxiety-SBP shared genetic loci of particular interest include BCKDK, KAT8, and LINC02210, which exhibit opposing effect directions on the two traits, whereas SAE1 and ATXN1L demonstrate consistent effect directions. Additionally, CNNM2 demonstrated consistent risk-enhancing effects across both NM (OR=1.25) and CAD (OR=1.94).

### Gene function exploration

For the genes highlighted in Fig 5, we performed comprehensive searches in the TWAS Atlas, IMPC, and MGI databases to investigate their potential associations with cardiovascular and mental health traits. Specifically, CNNM2 was found significantly associated with schizophrenia, SBP, and Pulse Rate (PR) in a TWAS analysis (Fig. 6 **A**). Compared to wild-type mice, CNNM2 knockout mice exhibit cardiovascular phenotypes, including altered heart weight, heart rate (HR), respiratory rate (RR), and pulse rate (PR). Additionally, they display neurological phenotypes such as changes in anxiety levels, motor abilities, exploratory behavior, and responses to environmental stimuli. (Fig. 6 **D**). Additionally, pathogenic mutations in CNNM2 are associated with cerebrovascular defects (such as hemorrhage) and neurodevelopmental malformations, including spina bifida and exencephaly (Fig. 6 **E**).

**Figure 5:**
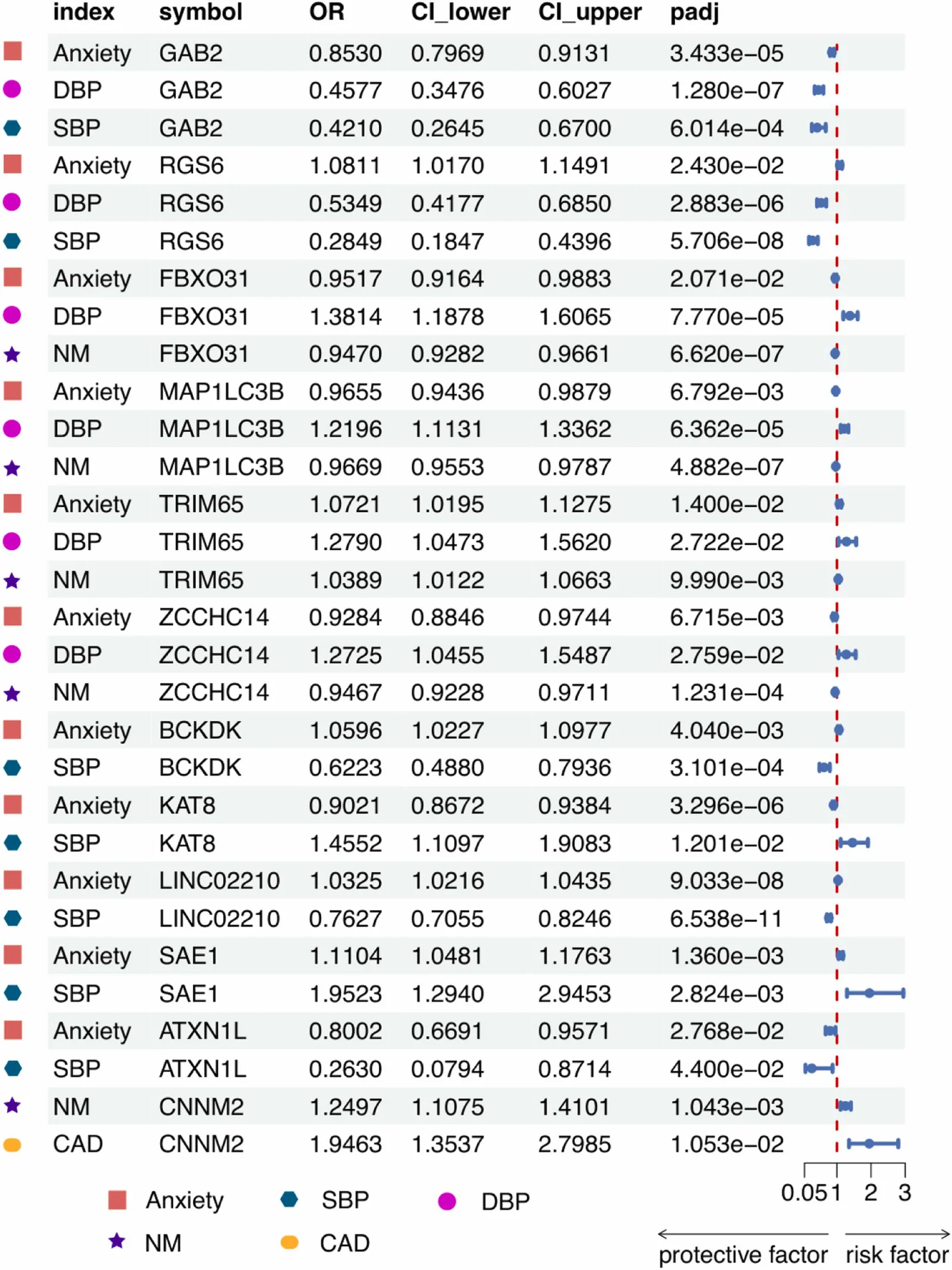
**Forest plot of the SMR analysis**. This figure presents the causal effects assessed by SMR analysis on phenotype pairs identified with causal relationships. Effect size estimates and their 95% confidence intervals are represented by horizontal lines (confidence intervals) and central points (point estimates).

**Fig. 6:**
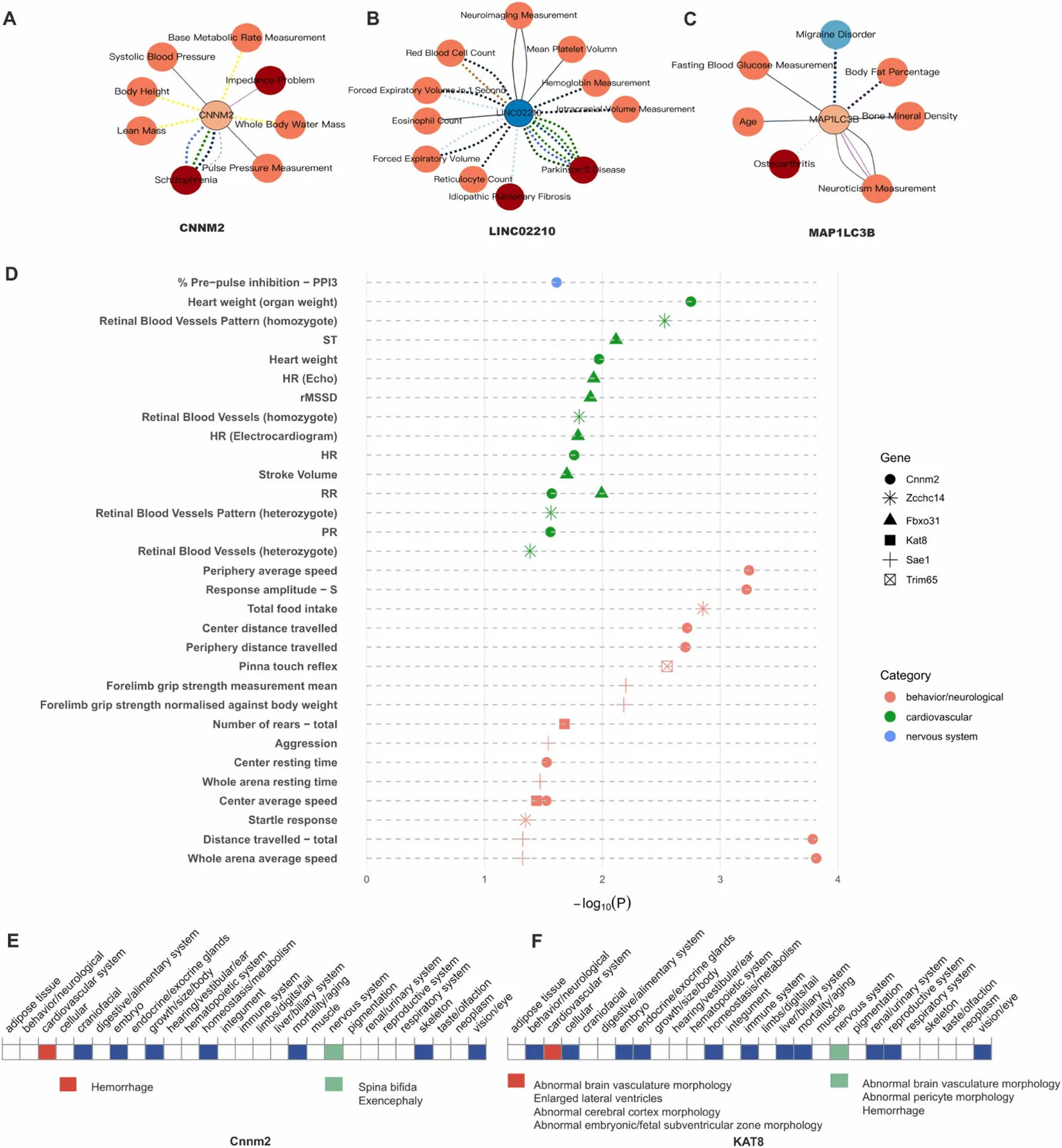
Functional annotation of pleiotropic genes across multi-omics platforms. (A–C) TWAS Atlas associations for CNNM2, LINC02210, and MAP1LC3B, respectively. (D) IMPC knockout phenotypes of candidate genes. (E–F) MGI phenotype annotations for Cnnm2 and Kat8, respectively.

TWAS analysis reveals that LINC02210 is associated with Parkinson's disease and intracranial volume abnormalities in the nervous system, as well as with red blood cell count and hemoglobin levels in the cardiovascular system (Fig. 6 **B**). MAP1LC3B is linked to migraine, neuroticism, and elevated fasting blood glucose levels (Fig. 6 **C**). Zcchc14 knockout mice exhibit associations with heart rate variability (rMSSD, RR interval) and abnormal feeding behavior (Fig. 6 **D**). Pathogenic mutations in KAT8 lead to cerebrovascular defects and abnormal development of the embryonic brain ventricles (Fig. 6 **F**).

## Discussion

Our study utilized LDSC analysis to explore the genetic correlation between CVS and MH traits. The results indicate that CVS traits generally exhibit higher heritability compared to MH traits. Notably, AN and ADHD showed the highest heritability estimates. Previous research demonstrates childhood ADHD heritability is 70–80%, which remains similarly high in adulthood (70–80%) using cross-informant approaches [27], highlighting genetic stability. In contrast, the heritability estimates for SBP and DBP were relatively lower, which may reflect a more balanced influence of genetic and environmental factors on these traits. For example, large-scale cohort studies have shown a significant association between healthy lifestyle scores and blood pressure as well as cardiovascular disease events, independent of individuals' genetic risk [28]. This finding suggests that even in populations with high genetic susceptibility, environmental interventions (such as lifestyle improvements) may still play a decisive role in blood pressure regulation.

Moreover, the genetic correlation analysis revealed varying degrees of overlap between CVS and MH traits. Specifically, MI demonstrated the most extensive genetic overlap with MH phenotypes. This finding is consistent with existing results, which highlights the frequent co-occurrence of mental disorders in patients with acute MI [29]. For instance, a large-scale study involving 6117,804 MI-hospitalized patients [30] found that major depression and anxiety each accounted for approximately 6% of the complications, followed by bipolar disorder, schizophrenia, and post-traumatic stress disorder. These results lend further support to our findings, underscoring the complex interplay between cardiovascular and MH conditions.

Although the effect of DBP on anxiety was relatively modest (OR = 1.08), anxiety exhibited a markedly stronger causal effect on DBP (OR = 1.4). This observation suggests that anxiety may not merely arise as a consequence of dysregulated blood pressure but could instead act as a potential driver of blood pressure alterations, particularly in DBP. These findings align with clinical investigations demonstrating associations between anxiety and circadian blood pressure rhythm abnormalities in hypertensive patients. For instance, heightened anxiety levels correlate significantly with aberrant blood pressure fluctuations during the morning surge period [31]. A two-year prospective cohort study further demonstrated that participants exhibiting baseline anxiety symptoms had a significantly elevated risk of developing incident hypertension [32], further supporting the hypothesis that anxiety exacerbates hypertension.

Additionally, through SMR analysis, we identified two genes (RGS6 and GAB2) that simultaneously influence anxiety and blood pressure. This finding further suggests the existence of shared biological mechanisms between neuropsychiatric disorders and CVD. RGS6 encodes a protein that is a member of the RGS (Regulator of G Protein Signaling) family, which plays a crucial role in modulating G protein-coupled receptor signaling pathways [33]. Previous studies have shown that RGS6 exerts anxiety-inducing and depressive effects by inhibiting serotoninergic innervation in the cortical-limbic neuronal circuits [34]. RGS6-deficient mouse models demonstrate significantly enhanced sympathetic nervous activity, accompanied by a trend toward elevated blood pressure [35], providing crucial animal-based evidence to understand the role of RGS6 in the relationship between anxiety and hypertension. More importantly, a population-based study indicated that G-protein signaling dysregulation mediated by RGS6 might exacerbate nocturnal hypertension, particularly in individuals with coexisting sleep disorders [36]. This suggests that RGS6 plays a critical role not only in the regulation of anxiety symptoms but also in controlling blood pressure through the modulation of sympathetic nervous system activity, thereby influencing the development of cardiovascular diseases.

Building upon the genetic evidence linking anxiety to cardiovascular regulation, we further explored NM may serve as phenotypic bridges between psychiatric vulnerability and cardiovascular pathogenesis. Our results found NM is highly prevalent in psychopathological research, with NM reflecting an individual's characteristic (or average) level of distress over an extended period [37]. We found a 1 s.d. increase in NM was tied to a 48.97% higher odds of CAD (OR = 1.4484). It is worth noting that the study suggests a link between neuroticism and increased somatic complaints, including chest pain or angina-like discomfort, but not with the objective signs or pathophysiological evidence of diseases (especially CAD) [38]. This suggests that the interplay between psychological and biological factors merits further investigation in the context of cardiovascular disease risk. Additionally, NM is also positively correlated with MI and AP. Participants characterized by diligent and sociable personalities were less prone to myocardial infarction, whereas those with neurotic traits exhibited notably higher risks [39].

Further SMR analysis identified several genes (e.g., CNNM2, KAT8, GAB2) whose expression levels in peripheral blood were causally associated with cardiovascular and neuropsychiatric phenotypes. For instance, CNNM2, a critical regulator of magnesium ion transport [40], was significantly associated with increased risk of NM (OR = 1.25) and the incidence of coronary artery disease (OR = 1.95). Our functional annotations further support the cross-system effects of this candidate gene. For instance, Cnnm2 knockout mice exhibit increased heart weight, retinal vascular pattern disruption, and abnormal locomotor behavior, which closely align with its associations with systolic blood pressure and schizophrenia observed in human TWAS. Recent studies have indicated that mutations in CNNM2 are linked to intellectual disabilities, seizures, and hypomagnesemia, highlighting its essential role in brain development, neuronal function, and magnesium homeostasis [41]. Additionally, GWAS have elucidated the pleiotropic nature of this locus: common variants within the CNNM2 gene are associated with neuropsychiatric disorders such as schizophrenia and bipolar disorder [42], [43], as well as cardiovascular phenotypes, including blood pressure, coronary artery disease, and myocardial infarction [44], [45], [46]. Collectively, the diverse physiological roles and pleiotropic effects of CNNM2 are consistent with it being a candidate pleiotropic gene linking neuropsychiatric and cardiovascular pathways, warranting functional validation in future studies. Similarly, KAT8, a histone acetyltransferase involved in cerebrovascular abnormalities and defects in brain ventricle development, may regulate both angiogenesis and neuroprogenitor cell differentiation through epigenetic mechanisms, such as H4K16 acetylation [47], [48]. These results underscore the pleiotropic effects of gene expression in the pathogenesis of cardiovascular-neuropsychiatric comorbidity.

Several additional genes identified in our analysis may also be relevant. FBXO31 has been implicated in endothelial senescence in atherosclerosis [49], whereas TRIM65 has been reported to promote vascular smooth muscle cell phenotypic switching through PI3K/Akt/mTOR signaling [50]. These findings suggest that both genes may be involved in vascular dysfunction, although through different cellular processes. ZCCHC14 has been associated with small-vessel stroke [51] and with nicotine- and alcohol-related traits [52], suggesting possible relevance to both cerebrovascular and behavioral phenotypes. BCKDK regulates branched-chain amino acid catabolism. Loss of BCKDK function has been linked to seizures and repetitive behavioral abnormalities, while altered branched-chain amino acid metabolism has also been reported in cardiovascular and metabolic disorders [53]. These studies provide biological context for the genes identified here, but their roles in the shared genetic basis of cardiovascular and mental health traits remain unclear.

In summary, our findings underscore the importance of recognizing the intertwined relationship between psychological traits and cardiovascular health at the genetic level. As this study is based on genetic association data without experimental or clinical validation, these findings should be interpreted as hypothesis-generating rather than as a basis for specific clinical or therapeutic recommendations. Moreover, as all GWAS data analyzed here were derived from European-ancestry populations, these associations may not generalize to other populations, and cross-ancestry replication will be an important direction for future work. Further functional and experimental research is needed to unravel the underlying mechanisms and to determine whether these shared genetic pathways have practical relevance for the clinical management of these comorbid conditions.

## Ethics approval and consent to participate

Not applicable.

## Funding

This research was funded by Taishan Scholars Construction Engineering, 10.13039/501100001809National Natural Science Foundation of China (32170989), Major Basic Research Project of Shandong Provincial Natural Science Foundation (ZR2019ZD27), 10.13039/100014103Key research and development program of shandong province (2023CXPTO12), 10.13039/501100007129Natural Science Foundation of Shandong Province (ZR2021MH141, ZR2023MH373), Binzhou Medical University Research Start-up (50012305190). This work was also supported by the Special Funding for the "Case-by-Case Introduction of Top Talent (Teams)" Program in Yantai. Social Science Planning Project of Shandong Province(Grant No.23CJYJ02).

## CRediT authorship contribution statement

Conceptualization, Jia Mi, Chunhua Yang, and Fuyi Xu; Formal analysis, Quanting Yin, Jun Zhao; Funding acquisition, Jia Mi, Fuyi Xu, Chunhua Yang, Geng Tian; Investigation, Pengcheng Yi; Methodology, Fuyi Xu; Supervision, Fuyi Xu; Validation, Quanting Yin; Visualization, Quanting Ying; Writing—original draft, Fuyi Xu, Jun Zhao and Quanting Yin; Writing—review and editing, Chunhua Yang, Jia Mi, Fuyi Xu, Li Shang, Qingxia He, Geng Tian.

## Declaration of Competing Interest

The authors declare that they have no competing interests.

## Footnotes

Appendix A Supplementary data associated with this article can be found in the online version at doi:10.1016/j.gmg.2026.100122.
