# Supplementary material for: Cross-trait analysis reveals shared genetic architecture between mental health and cardiovascular traits
Source: Glob Med Genet. 2026 Jul 24;13(4):100122. doi: 10.1016/j.gmg.2026.100122 (PMC13677588; doi:10.1016/j.gmg.2026.100122)
Supplement: Supplementary file 1 [file mmc2.pdf]

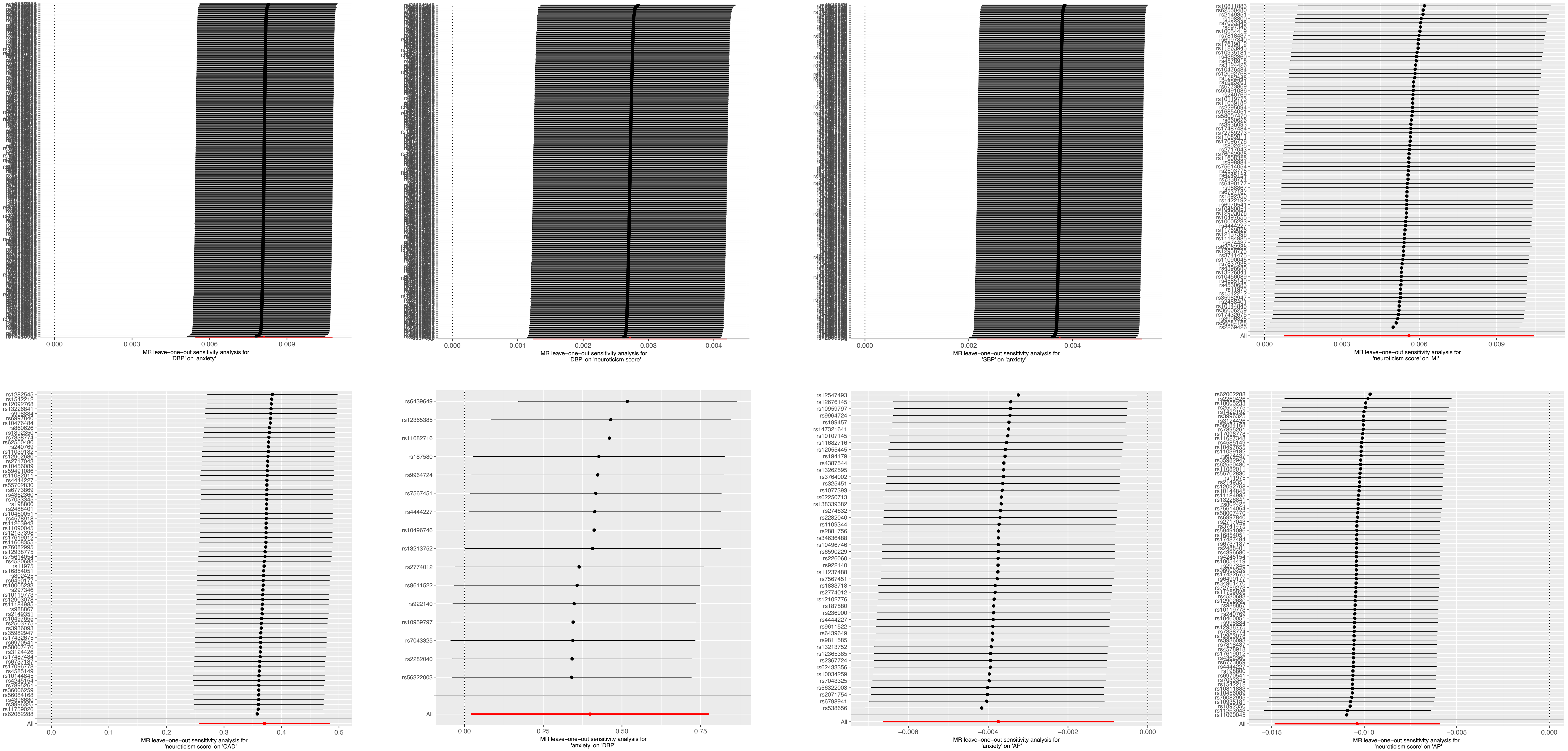

0.000
0.003
0.006
0.009
MR leave−one−out sensitivity analysis for
'DBP' on 'anxiety'
0.000
0.001
0.002
0.003
0.004
MR leave−one−out sensitivity analysis for
'DBP' on 'neuroticism score'
0.000
0.002
0.004
MR leave−one−out sensitivity analysis for
'SBP' on 'anxiety'
All
0.000
0.003
0.006
0.009
MR leave−one−out sensitivity analysis for
'neuroticism score' on 'MI'
All
0.0
0.1
0.2
0.3
0.4
0.5
MR leave−one−out sensitivity analysis for
'neuroticism score' on 'CAD'
rs6439649
rs12365385
rs11682716
rs187580
rs9964724
rs7567451
rs4444227
rs10496746
rs13213752
rs2774012
rs9611522
rs922140
rs10959797
rs7043325
rs2282040
rs56322003
All
0.00
0.25
0.50
0.75
MR leave−one−out sensitivity analysis for
'anxiety' on 'DBP'
All
−0.006
−0.004
−0.002
0.000
MR leave−one−out sensitivity analysis for
'anxiety' on 'AP'
All
−0.015
−0.010
−0.005
0.000
MR leave−one−out sensitivity analysis for
'neuroticism score' on 'AP'
